# Dominance of photo over chromatic acclimation strategies by habitat-forming mesophotic red algae

**DOI:** 10.1098/rspb.2023.1329

**Published:** 2023-10-04

**Authors:** Sofie E. Voerman, Beauregard C. Marsh, Ricardo G. Bahia, Guilherme H. Pereira-Filho, Ana Clara F. Becker, Gilberto M. Amado-Filho, Arvydas Ruseckas, Graham A. Turnbull, Ifor D. W. Samuel, Heidi L. Burdett

**Affiliations:** ^1^ Lyell Centre for Earth and Marine Science and Technology, Edinburgh, UK; ^2^ School of Energy, Geoscience, Infrastructure and Society, Heriot-Watt University, Edinburgh, UK; ^3^ Botanical Garden Research Institute of Rio de Janeiro, Rio de Janeiro, Brazil; ^4^ Laboratório de Ecologia e Conservação Marinha, Instituto do Mar, Universidade Federal de São Paulo, Santos, São Paulo, Brazil; ^5^ Organic Semiconductor Centre, SUPA, School of Physics and Astronomy, University of St Andrews, St Andrews, UK; ^6^ Umeå Marine Sciences Centre, Umeå University, Norrbyn, Sweden; ^7^ Department of Ecology & Environmental Science, Umeå University, Umeå, Sweden

**Keywords:** maerl, rhodolith, mesophotic reef, photosynthesis, pam fluorometry

## Abstract

Red coralline algae are the deepest living macroalgae, capable of creating spatially complex reefs from the intertidal to 100+ m depth with global ecological and biogeochemical significance. How these algae maintain photosynthetic function under increasingly limiting light intensity and spectral availability is key to explaining their large depth distribution. Here, we investigated the photo- and chromatic acclimation and morphological change of free-living red coralline algae towards mesophotic depths in the Fernando do Noronha archipelago, Brazil. From 13 to 86 m depth, thalli tended to become smaller and less complex. We observed a dominance of the photo-acclimatory response, characterized by an increase in photosynthetic efficiency and a decrease in maximum electron transport rate. Chromatic acclimation was generally stable across the euphotic-mesophotic transition with no clear depth trend. Taxonomic comparisons suggest these photosynthetic strategies are conserved to at least the Order level. Light saturation necessitated the use of photoprotection to 65 m depth, while optimal light levels were met at 86 m. Changes to the light environment (e.g. reduced water clarity) due to human activities therefore places these mesophotic algae at risk of light limitation, necessitating the importance of maintaining good water quality for the conservation and protection of mesophotic habitats.

## 1. Background

The mesophotic oceanic zone (typically approx. 30–150 m water depth) harbours diverse and unique benthic ecosystems created by ecosystem engineers such as corals and macroalgae that provide global socio-ecological benefits [1]. Given the reliance of these engineers on sunlight for energy demands, it was long assumed that their distribution was limited to the shallow euphotic coastal zone [2]. However, technological advancements have enabled us in recent decades to confirm their growth well into the mesophotic zone. While corals are able to shift towards higher heterotrophic reliance [3], photosynthetic acclimation is fundamental to mesophotic macroalgal success but the acclimatory strategies remain poorly defined [4].

With increasing ocean depth, several environmental changes may be observed, including reduced irradiance, narrowing of the light spectral range, reduced temperature and altered water flow. These significantly affect the ecological functioning of macroalgal ecosystem engineers [5] (particularly in their morphology [e.g. 6]), reflecting a requirement to regulate light acclimation, maximize energy provision and tolerate environmental gradients. This can alter the quantity and quality of biogenic habitat, with follow-on impacts for the diversity and composition of associated communities and the higher ecosystem services [e.g. 7]. Changes in the light field are therefore crucial for the functioning of benthic photoautotrophs in the mesophotic [e.g. 8,9] and their wider associated ecosystems.

However, photosynthesis is not equally driven by all wavelengths within the range of photosynthetically active radiation (PAR, 400–700 nm) [10] so the type of light-harvesting complexes dictates the quality of available irradiance for photosynthesis [5]. Non-geniculate red coralline algae (Florideophyceae, Rhodophyta) are the deepest known benthic macroalgae, with records at 300+ m [11]. The capacity for red algae to survive under mesophotic conditions has been attributed to the presence of phycobilisomes [12]—multi-pigment light-harvesting complexes that funnel photon energy towards the photosystems. Phycobilisomes strongly absorb in the intermediate PAR range that becomes dominant with ocean depth, filling a gap where chlorophyll absorbs poorly and maximizing photon capture in an environment of increasingly low photon availability [5]. Flexibility in the ratios of the key phycobilisome pigments phycoerythrin and phycocyanin allow for fine-scale regulation of their absorption spectra (i.e. chromo-acclimation), tailored to the *in situ* light environment [13,14].

In their free-living form, coralline algae create diverse reef ecosystems (called maerl/rhodolith beds) with a cosmopolitan distribution from the poles to the tropics and from intertidal to mesophotic depths [15] that have globally significant roles in biodiversity provision [7] and biogeochemical cycling [16–18]. Despite their ecological importance, understanding of the photosynthetic processes that underpin their survival is only recently emerging. In shallow-water high-light environments, free-living coralline algae have a high capacity for photo- and chromo-acclimation to spatial and temporal variations in light availability [14,19]. However, shallow-water specimens are consistently light saturated, suggesting that photosynthetic optimization may be achieved in the deep euphotic or mesophotic zones. To investigate this, we present the first investigation of photosynthetic acclimation of mesophotic free-living coralline algae. By comparing across a 13–86 m depth range, we identify a shift in acclimatory strategy with depth independent of taxonomic classification, providing new insights into free-living coralline algal physiology, which fundamentally underpins their capacity to support high biodiversity and biogeochemical activity.

## 2. Material and methods

### (a) Sampling location

Free-living coralline algal thalli and the *in situ* light environment were sampled at five depths spanning a 73 m depth gradient (13, 40, 56, 65 and 86 m deep) from five different locations around the Fernando de Noronha archipelago (electronic supplementary material, figure S1) across a 5-day period in September/October 2018.

### (b) *In situ* light environment

The *in situ* light environment at each sampling location was quantified immediately after algal sampling using a SATLANTIC OCR-507 Multispectral Radiometer (OCR) in combination with an OCEAN SEVEN 304 CTD between 11.00 and 13.00 on the day of sampling, with full-sun conditions and a calm sea state. Irradiance intensity was measured across seven discrete 10 nm width bands within the PAR range (peaks at 412.6, 443.6, 489.7, 532.1, 554.7, 664.8 and 683.5 nm). Total PAR at the seabed was calculated via a linear interpolation across the OCR measurement bands and extrapolation from the lower limit of the deployment profiles using a logarithmic model of the depth profiles (electronic supplementary material, table S1), validated via cross-comparison to the co-deployment of an Odyssey PAR logger (depth-limited to 30 m; calibrated against an Apogee quantum sensor). Due to malfunctioning of the CTD at the 86 m site, extrapolation from 65 m was used.

The diffuse attenuation coefficient for downward plane PAR (K_d_PAR) was estimated following [5]:

$$E[z]=E[0]*\exp(-K_{\mathrm{dPAR}}*z),$$

where *E*[*z*] is the irradiance at a given depth *z* and *E*[0] is irradiance at the water surface.

### (c) Free-living coralline algal sampling

At each depth, free-living coralline algal thalli (*n* = 18 individuals per depth) were hand-collected using SCUBA. Thalli were non-intentionally targeted in intervals of five diver fin kicks, apart from at 86 m where all samples were collected within a 4 m^2^ area due to bottom time restrictions. Thalli covered by large fleshy algae (e.g. Dictyotales) were avoided. All thalli were returned to the surface within 1 hour of collection and stored in the dark at ambient water temperature (approx. 27°C) before transfer to shore.

### (d) Coralline algal species identification

Taxonomic identification of the thalli from each depth was based on morphoanatomical analyses using the histological methods described in [20] and comparison to known species of the region [21–24].

### (e) Coralline algal morphometrics

Thallus surface area, volume, sphericity and complexity were calculated from three-dimensional scans of thalli from each depth with approximately 100 µm point resolution using a laser scanner and accompanying ScanStudio software (NextEngine, Inc., USA). Lengths of the *x*,*y*,*z* axes were used to calculate sphericity, following [25]. Surface complexity was calculated as the average of the three-dimensional surface area of three randomly chosen 1 cm^2^ areas from each scanned thallus.

### (f) Algal photosynthetic characteristics

Photosynthetic characteristics of the coralline algal thalli were measured using pulse amplitude modulation (PAM) fluorometry, conducted within 2 h of collection. Rapid light curves (RLCs) were conducted using the blue-light Junior PAM (Walz GmbH), following previous methodologies and notations [14,19] (electronic supplementary material, table S2) using a 2 mm diameter fibre optic probe positioned directly on the surface of the branch tip, held flat against the surface with a clamp. RLCs were performed in the dark to ensure initial dark adaptation and calculation of maximum photosynthetic efficiency (*F*_v_/*F*_m_), with the following PAM settings: measuring light intensity = 8, gain = 1, saturation pulse intensity = 12, saturation pulse width = 0.6, actinic light intensity = 5, with eight irradiance steps of 10 s duration ranging from 0 to 420 µmol photons m^−2^ s^−1^. Minimum saturating intensity (*E*_k_—the irradiance level (µmol photons m^−2^ s^−1^) at which light shifts from being photosynthetically limiting to photosynthetically saturating) and initial photosynthetic rate (alpha [*α*]; no units) were calculated by fitting RLC data to the irradiance-normalized nonlinear least-squares regression model of Jassby & Platt [26] in the R package Phytotools [27] to describe the light response of quantum efficiency using the following equation:

$$y=\left(\frac{1}{x}\right)*\alpha *E_{k}*\tan h\left(\frac{x}{E_{k}}\right),$$

where *x* = PAR level at a given RLC step and *y* = *F*_q_'/*F*_m_' the RLC step. All model fits were statistically significant (model *p*-value less than 0.0001 for all).

### (g) Algal pigment composition

Immediately following RLC measurements, optical reflectance of the surface of each individual was determined as a measure of tissue pigment composition following an adapted method from [14,28]. A Flame-S-VIS-NIR Miniature Spectrometer was used, equipped with a high-power tungsten halogen light source (HL-2000-HP-FHSA; 360–2400 nm) and 400 µm laboratory-grade reflection probe to collect reflected light (Ocean Optics). An integration time of 2500 µs was used with a boxcar width of one. The spectrometer probe was maintained at a 45° angle for all measurements to measure diffuse reflectance and avoid specular light effects that can occur when holding the probe perpendicular to a sample. Following the manufacturer's recommendation, the probe was held 4 mm from each sample to maximize the efficiency of the overlapping illuminating fibres. The spectrophotometer was calibrated using a white reflectance standard (Ocean Optics) that represents 100% reflectance and a dark calibration to account for any ambient light before each set of measurements (measurements were conducted in the dark to minimize ambient light). Absorbance was calculated following [29] and [30]:

$$D(\lambda )=\log\left[\frac{1}{R(\lambda )}\right],$$

where *D* = absorbance, *λ* = wavelength and *R* = reflectance measurement. Absorbance was corrected for non-pigment absorption by subtracting the average absorbance between 750 and 800 nm. Transmission absorbance spectra showed the typical features of Rhodophyta (e.g. [28]) (see electronic supplementary material, figure S3), allowing us to use known pigment-specific maximum absorbance peaks as a proxy for relative pigment abundance: chlorophyll-a (680 nm), phycocyanin (630 nm) and phycoerythrin (495 nm), obtained from the absorption spectra. At 590–640 nm, there is an overlap between chlorophyll-a (30% contribution) and phycocyanin (70% contribution) [28], so a 0.7 correction factor was applied to the phycocyanin absorbance.

### (h) Statistical analysis

General linear mixed models were used to identify the effect of depth on photosynthetic characteristics, pigment composition and morphology. Where necessary, data were transformed to meet parametric assumptions. Depth was considered as a fixed factor, while algal taxonomy (and its interaction with depth) was considered as a random effect. To avoid overfitting, an interactive term was not included when this resulted in near singularity of the model. Where significant depth effects were found, *post hoc* Tukey test comparisons were used to determine differences between depths. Analyses were conducted in R version 4.1.1 [31], using the packages ‘lme4' [32] and ‘lmerTest' [33].

## 3. Results

### (a) *In situ* light environment

**Figure 1.**
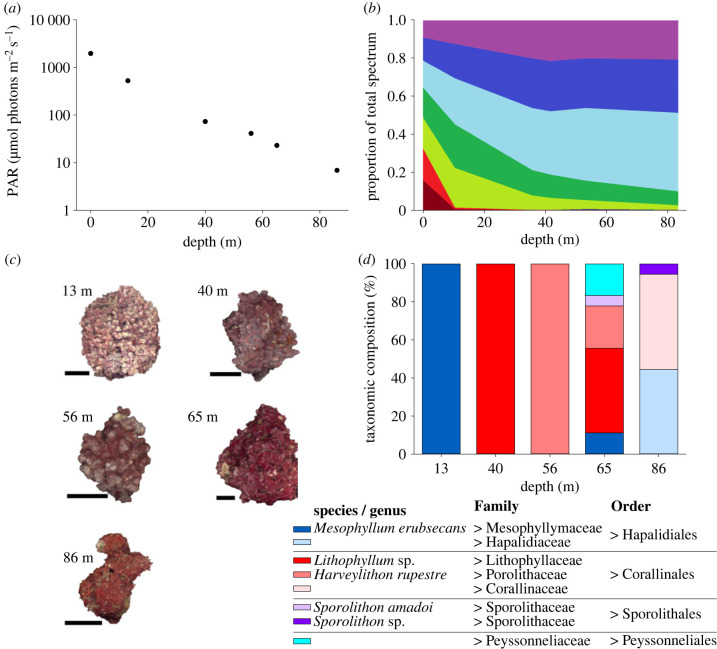
*In situ* light environment and coralline algal morphology and taxonomy from 13 to 86 m depth. (*a*) Photosynthetic active radiation (PAR) (400–700 nm) based on seabed extrapolation from depth profiles (see Methods and electronic supplementary material, table S1), (*b*) average spectral composition based on cumulative wavelength-specific irradiances, (*c*) example thalli from each sampling depth (scale bar = 2 cm) and (*d*) coralline algal species composition (% of all samples); individual thalli were visually confirmed to be composed of only a single taxon. Colour tones on bars indicate Order classification; identification to species level was conducted where possible (i.e. when reproductive structures were present). *N* = 18 individual thalli were sampled per depth but should not be considered representative of the complete coralline algal species diversity, which is presented in Amado-Filho *et al.* [22].

Midday PAR declined exponentially with depth, from 1965 µmol photons m^−2^ s^−1^ at the surface to 6.5 µmol photons m^−2^ s^−1^ at 86 m depth – 0.33% of surface irradiance (figure 1*a*). The attenuation coefficient K_d_PAR was 0.068 m^−1^. PAR at the 30 m depth definition of mesophotic was 255 µmol photons m^−2^ s^−1^, compared with an estimated depth of 78 m for the 1% of surface irradiance (i.e. 20 µmol photons m^−2^ s^−1^) definition for macroalgal growth limits [2]. Spectrally, there was a rapid loss in the long wavelength region (665 and 684 nm) in the upper 13 m of water column, and represented less than 1% of the total PAR at the seabed at each depth (figure 1*b*). At 40 m depth, there was a decline in the relative contribution of the mid-long wavelength bands (532 and 555 nm) to less than 10% of the overall spectrum at 86 m depth. Short wavelength bands (413 and 444 nm) comprised approximately 20% each from 40 m depth. The mid-short wavelength band (490 nm) increased in relative proportion from less than 20% at the surface to greater than 40% at 86 m depth (figure 1*b*).

### (b) Coralline algal context

Although the data should not be considered the complete coralline algae diversity at each depth (see [22] for a dedicated biodiversity study), a range of coralline algae taxa are represented. Visually, each thallus was composed of a single species, with specimens collected from seven coralline algae Families: Mesophyllumaceae, Lithophyllaceae, Porolithaceae, Corallinaceae, Sporolithaceae and Peyssonneliaceae (figure 1*c,d*). No significant taxonomic effects were observed for any of the morphological nor photosynthetic parameters (electronic supplementary material, tables S3–S5). By including taxon (as Family) as a random predictor, the observed depth effects (detailed below) take taxon identity into account.

**Figure 2.**
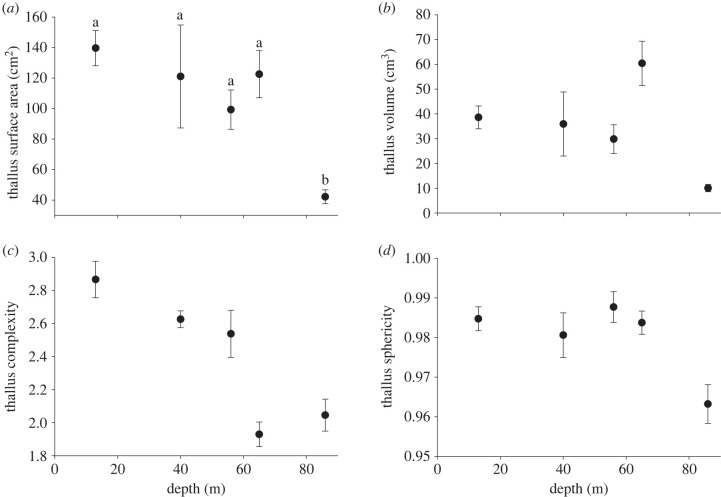
Morphometric traits of free-living coralline algal thalli from 13 to 86 m depth, pooled across levels of Family. Thallus (*a*) surface area, (*b*) volume, (*c*) complexity and (*d*) sphericity. Data presented as mean ± s.e., *N* = 10, 5, 5, 16, 15 for depths 13, 40, 56, 65 and 86 m, respectively. Letters above symbols indicate statistically different depth groupings (cf. electronic supplementary material, table S4).

Irrespective of taxonomic classification, all coralline algal morphometrics measured were generally lower at deeper depths, suggesting the mesophotic coralline algae beds were composed of smaller, less complex and flatter thalli. However, this was statistically significant only for surface area, where a minimum was observed at 86 m depth (figure 2; electronic supplementary material, table S3).

### (c) Photo-acclimation with depth

**Figure 3.**
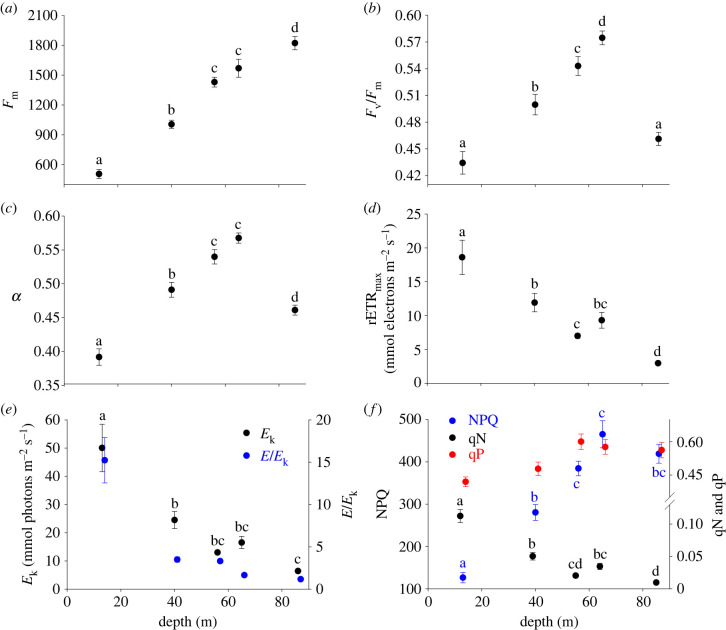
Photosynthetic characteristics of free-living coralline algal thalli from 13 to 86 m depth, pooled across taxonomic Family. (*a*) Maximum fluorescence (*F_m_*), (*b*) maximum quantum yield (*F_v_/F_m_*), (*c*) photosynthetic efficiency in light-limiting conditions (*α*), (*d*) light-saturated electron transport (rETR_max_), (*e*) saturating irradiance (*E_k_*) and proportion of total PAR (*E*/*E_k_*) (symbols jittered for clarity) and (*f*) non-photochemical (as NPQ and 1-qN) and photochemical quenching (qP). Letters associated with symbols (colour-coded to match symbol colour) indicate statistically different groupings (cf. electronic supplementary material, table S3). Error bars represent mean ± s.e. *N* = 18 per depth; where error bars are not visible they are smaller than the symbol. See electronic supplementary material, table S2 for photosynthetic characteristic definitions.

Taking taxonomic classification into account, depth still had a significant effect on all photosynthetic characteristics except qP (figure 3; electronic supplementary material, table S4). *F*_m_ significantly increased from shallow to deeper depths (figure 2*a*; electronic supplementary material, table S4). Photochemical efficiency (as measured in *F*_v_/*F*_m_ and *α*) significantly increased from 13 to 65 m depth, followed by a sharp decline at 86 m depth (figure 2*b,c*; electronic supplementary material, table S4). Conversely, both rETR_max_ and *E*_k_ significantly decreased from 13 to 86 m, but with a slight increase at 65 m (figure 2*d,e*; electronic supplementary material, table S4). At 86 m depth, *E*_k_ was close to the ambient PAR levels (indicating optimal photon availability), compared with greater than 15x excess photon availability at 13 m depth (figure 2*e*; electronic supplementary material, table S4). Non-photochemical quenching (as NPQ and 1-qN) was lowest at 13 m, and significantly higher at deeper depths. This was accompanied by the lowest photochemical quenching (qP) at 13 m (figure 3*f*; electronic supplementary material, table S4).

### (d) Chromatic acclimation with depth

**Figure 4.**
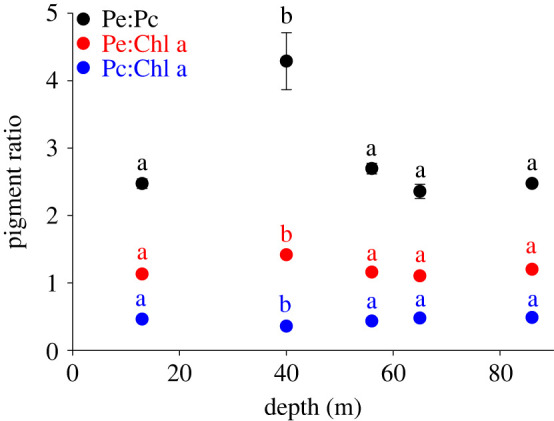
Absorbance-derived pigment ratios of free-living coralline algal thalli from 13 to 86 m depth, pooled across taxonomic Family. Pe, phycoerythrin (*λ*max = 495 nm); Pc, phycocyanin (*λ*max = 630 nm) and Chla: chlorophyll-a (*λ*max = 680 nm). Letters above symbols indicate statistically different groupings (cf. electronic supplementary material, table S4). Error bars represent mean ± s.e. *N* = 18 per depth; where error bars are not visible they are smaller than the symbol. Complete absorbance spectra from 400 to 700 nm are provided in electronic supplementary material, figure S2.

Taking taxonomic classification into account, relative pigment composition was significantly affected by depth (electronic supplementary material, table S5). This was driven by a significant difference in pigment ratios at 40 m depth (figure 4), including significantly higher Pe:Pc and Pe:Chl a ratios compared with the other depths, and a significantly lower Pc:Chl a compared with the other depths.

## 4. Discussion

Photosynthetic persistence in the mesophotic is dependent on the quantity and quality of available light and a species' acclimatory capacity to maximize light utilization. Here, we present the first photo-acclimatory strategies of free-living red coralline algae across the euphotic-mesophotic transition, with taxonomic and morphological context that goes beyond previous work on mesophotic algal photosynthesis. We show that in the euphotic zone the photosynthetic apparatus is tuned to balance energy conversion, photodamage risk and resource allocation. In the mesophotic, this shifts towards prioritizing maximal light harvesting to enable a near-complete capture of available photons.

### (a) Morphological acclimation

Variations in organismal complexity is a common acclimatory mechanism adopted for optimizing photosynthesis across varying light environments [6,34]. In free-living coralline algae, complexity regulates the extent of self-shading and subsequent within-individual variation in photosynthetic acclimation [19,34]. This might explain the observed higher thallus complexity at shallower depths. In parallel, low energy assimilation due to reduced light availability will directly affect algal growth potential in the mesophotic [35,36], resulting in the observed smaller, less complex thalli in deeper waters. Transcriptomic and temporal growth reconstructions could help to identify whether morphological changes are driven by acclimation/adaptation to light availability, or as a result of differences in energy provision with depth as a limitation to growth. However, in contrast to the flattening of mesophotic corals with depth [3], variability in coralline algal thallus sphericity appears to be retained through the euphotic-mesophotic transition, enabling continuity of habitat provision [7].

Photophysiology measurements here were taken from branch tips and therefore not affected by gross morphological variations such as self-shading. Nevertheless, it is likely that intra-individual variation would have been identified if investigated. Our emerging understanding of photo- and chromo-acclimation in red coralline algae (here and e.g. [14,19,28]) necessitates the use of light-harvesting modelling to uncover how gross morphological change affects organismal-scale light capture, energy transfer and carbon assimilation. Although taxon-specific variations in photo-acclimatory capacity were not identified, this cannot be ruled out for the natural coralline algal community—further work specifically designed to address inter-taxon variability is required to ensure all species at all depths are considered.

### (b) Light intensity and photo-acclimation

Midday seabed irradiance from 13 to 86 m depth spanned two orders of magnitude, with an attenuation coefficient characteristic of clear oceanic waters (Jerlov type 1A, [37]). While seasonal differences in light penetration may occur between rainy and dry seasons, land run-off is low, resulting in negligible effects on variability in water column turbidity. The light profiles presented here are therefore considered to be more widely representative of typical light profiles around Fernando do Noronha, allowing us to interpret the photophysiological results as being broadly representative of the euphotic-mesophotic coralline algal photo-acclimation strategies. Maximum light levels at our deepest location were below that previously thought to be uninhabitable for benthic macroalgae (1% of surface irradiance [2]), and previous reports indicate further extension of coralline algal beds to 100+ m [22]. Using our K_d_PAR, maximum light levels at 100 m are expected to be approximately 2 µmol m^−2^ s^−1^ – 0.11% of surface irradiance.

In response to this, clear indicators for depth-driven photo-acclimation were observed at lower light levels, including an increase in *α*, a decrease in rETR_max_ and a decrease in the saturating irradiance (*E*_k_). This provides important field-based evidence to complement recent experimental definition of the light-harvesting pathway in red coralline algae [28]. Some caution must be given to PAM fluorescence interpretations, especially for the red algae, because of signal interactions from other pigments and a lack of corresponding photosynthetic rate measurements [19,28]. Nevertheless, our approach enabled us to maintain methodological consistency between RLCs (e.g. distance and angle of probe from the algal surface), and acquire considerably more thallus replicates than has previously been achieved. In contrast to previous work (e.g. [38]), this allows us to directly compare average responses and inter-individual variability across depths, gaining important new insight into the variability of the coralline algal photo-acclimatory response across the euphotic-mesophotic transition. At each depth, inter-individual variability was low—even when taxonomic diversity was comparatively high. This gives high confidence in the observed depth responses and the general interpretation of a euphotic-mesophotic shift in photo-acclimatory strategy.

The overall low observed *E*_k_ (less than 40 µmol photons m^−2^ s^−1^) supports the sciaphilic (shade-loving) nature of red coralline algae [19,39]. Under euphotic conditions, a balance between the energetic and resource cost of investing in light-harvesting apparatus (especially in nutrient-poor oceanic waters) and the potential carbon gain is adopted, evidenced by low *E*_k_ compared with the *in situ* PAR availability and the comparatively low photosynthetic efficiency (as *F_v_*/*F_m_* and *α*). Under mesophotic conditions, where the algae are operating almost at their functional limit (i.e. *E* / *E*_k_ ∼ 1), this balance shifts towards an acclimatory set-up designed to maximize photon harvesting. The proportion of absorbed photons relative to the total available must increase as depth increases—as has been previously observed in Antarctic under-ice cyanobacterial mats [40]. This implies that near-perfect photon capture will be required, to balance against continued carbon losses (e.g. respiration, carbonate dissolution and/or skeletal grazing). Given the success of free-living coralline algae throughout the euphotic and mesophotic zones of Fernando do Noronha, it is reasonable to assume that this photo-optimization is achieved in the long-term, supported by the recent identification of 94% phycobilisome energy transfer efficiency in red coralline algae [28]. Lower diel-scale photon availability in the mesophotic may explain the reduced quantum yield observed at 86 m, supporting an inhibition of *α* when conditions become challenging [41,42].

### (c) Spectral composition and chromo-acclimation

Downwelling benthic irradiance became increasingly dominated by shorter wavelengths with depth, consistent with an oceanic water type [37]. At this spectral range, while chlorophyll absorption is at a minimum, phycobilisome absorption is highly efficient [5]. Phycobilisome-containing organisms, such as red coralline algae, therefore hold a competitive advantage for photon harvesting in the mesophotic. However, no clear trends in pigment composition were observed with depth, supporting an *in situ* dual function for phycobilisomes as both efficient light-harvesting complexes in low light [12,28] and valuable photoprotective cascades in high light [12,43]. Similarly, specimens from the shallower depths were light saturated (*E* / *E*_k_ ≫ 1), but exhibited no clear change in pigment ratios. This is in contrast to cyanobacterial mats in ice-covered lakes, where phycobilin : chlorophyll-a ratios can increase with depth [40]. In red coralline algae, diel-scale temporal chromo-acclimation has been previously observed in high light shallow-water tropical reefs [14] (where midday PAR was almost twice that of the 13 m depth PAR in this study), and pigment reduction (bleaching) in encrusting coralline algae has been reported following removal of a kelp canopy [44]. Chromo-acclimation may therefore only become employed by coralline algae in high light environments or during light-shock events, suggesting its use as a photoprotective strategy rather than for maximizing energy transfer.

### (d) Ecosystem implications

Here, we provide insight into algal light-harvesting acclimation in the mesophotic zone—fundamental processes that underpin the formation of coralline algal beds and the services they can provide. This physiological understanding is therefore crucial for explaining and predicting trends such as the observed euphotic-mesophotic transition in coralline algal habitat biodiversity [7,45]. The low light availability at mesophotic depths means that photon capture and subsequent energy transfer must be close to 100% efficient to meet energy demands and to offset physiological (e.g. respiration, carbonate dissolution) and ecological (e.g. grazing) carbon losses. Although no direct evidence of coralline grazing in Fernando do Noronha is available, coralline algal grazing has been reported elsewhere (e.g. [46]) and may stimulate higher primary production and calcification rates [47]. Recent macrofaunal biodiversity surveys [7,45] reveal the presence of known coralline algal grazers—gastropods and echinoderms appear to be highest at the 56 and 65 m depths [7]. Drivers affecting faunal associations and habitat trophic cascades (e.g. changing climates, stock extraction) may therefore play an important role in the coralline algal photo-acclimatory strategy that ultimately underpins this ecosystem. Long-term reduction in light availability, or a shift in the spectral composition, may additionally place these algae into light limitation (especially in the mesophotic). If this is beyond the threshold for photo-acclimation, their long-term survival may be questioned. Around the world, coralline algal beds interact and conflict with human activities that reduce light availability, such as seabed resource extraction, bottom trawling and catchment deforestation [48,49]. It is widely accepted these actions negatively affect the community structure of coralline algal beds. However, here we show that these actions may also risk upsetting the fine physiological balance maintained by the coralline algae that underpin the entire habitat. Such activities should therefore be of priority conservation concern, with management solutions applied across the euphotic and mesophotic coastal zones.

## Data Availability

The data are provided in electronic supplementary material [50].
